# 14-3-3 epsilon prevents G2/M transition of fertilized mouse eggs by binding with CDC25B

**DOI:** 10.1186/s12861-014-0033-x

**Published:** 2014-07-25

**Authors:** Cheng Cui, Xiuli Ren, Dajun Liu, Xin Deng, Xin Qin, Xiangyu Zhao, Enhua Wang, Bingzhi Yu

**Affiliations:** 1 Department of physiology, China Medical University, Shenyang 110001, Liaoning, China; 2 Department of nephrology, Shengjing Hospital of China Medical University, Shenyang 110004, Liaoning, China; 3 Department of biochemical and molecular biology, China Medical University, Shenyang 110001, Liaoning, China; 4 Institute of pathology and pathophysiology, China Medical University, Shenyang 110001, Liaoning, China

**Keywords:** 14-3-3ε, CDC25B, MPF, Fertilized mouse eggs

## Abstract

**Background:**

The 14-3-3 (YWHA) proteins are highly conserved in higher eukaryotes, participate in various cellular signaling pathways including cell cycle regulation, development and growth. Our previous studies demonstrated that 14-3-3ε (YWHAE) is responsible for maintaining prophase | arrest in mouse oocyte. However, roles of 14-3-3ε in the mitosis of fertilized mouse eggs have remained unclear. Here, we showed that 14-3-3ε interacts and cooperates with CDC25B phosphorylated at Ser321 regulating G2/M transition of mitotic progress of fertilized mouse eggs.

**Results:**

Disruption of 14-3-3ε expression by RNAi prevented normal G2/M transition by inhibition of MPF activity and leaded to the translocation of CDC25B into the nucleus from the cytoplasm. Overexpression of 14-3-3ε-WT and unphosphorylatable CDC25B mutant (CDC25B-S321A) induced mitotic resumption in dbcAMP-arrested eggs. In addition, we examined endogenous and exogenous distribution of 14-3-3ε and CDC25B. Endogenous 14-3-3ε and CDC25B were co-localized primarily in the cytoplasm at the G1, S, early G2 and M phases whereas CDC25B was found to accumulate in the nucleus at the late G2 phase. Upon coexpression with RFP–14-3-3ε, GFP–CDC25B–WT and GFP–CDC25B–S321A were predominantly cytoplasmic at early G2 phase and then GFP–CDC25B–S321A moved to the nucleus whereas CDC25B-WT signals were observed in the cytoplasm without nucleus accumulation at late G2 phase at presence of dbcAMP.

**Conclusions:**

Our data indicate that 14-3-3ε is required for the mitotic entry in the fertilized mouse eggs. 14-3-3ε is primarily responsible for sequestering the CDC25B in cytoplasm and 14-3-3ε binding to CDC25B-S321 phosphorylated by PKA induces mitotic arrest at one-cell stage by inactivation of MPF in fertilized mouse eggs.

## Background

Progression through the cell-division cycle requires phosphorylation events carried out by cyclin-dependent protein kinases (CDKs) and the activation of the CDKs is a central issue of cell cycle regulation. The WEE1/MYT1 protein kinases mediate inhibitory phosphorylation of CDK1 (CDC2) on tyrosine 15 (Tyr15) and threonine 14 (Thr14) [1]–[3], whereas dualspecificity phosphatases of the CDC25 (cell division cycle 25) family can dephosphorylate phosphotyrosine as well as phosphothreonine residues, therefore activating their physiological substrates, the CDKs [4],[5]. In mammalian cells, CDC25 phosphatase family, including CDC25A, CDC25B and CDC25C, have been identified and found to regulate the cell cycle [6],[7]. In hamster BHK21 cell, CDC25B can activate initially CDC2/Cyclin B, also named the maturation-promoting factor (MPF), which initiates mitosis through the activation of CDC25C [8]. Our previous studies demonstrated that dephosphorylation of Ser 149 and Ser321 of CDC25B in the G2 phase induced the activation of CDC25B, which can activate MPF efficiently and resume mitosis by the direct dephosphorylation of CDC2-Tyr15 in fertilized mouse eggs [9],[10]. These results support that CDC25B plays a critical regulatory role in G2/M progression in the mitosis.

The 14-3-3 proteins are highly conserved in eukaryotes binding to their phospho-serine and phospho-threonine-containing ligands to regulate a wide range of cellular phenomena involved in development and growth including cell cycle control, apoptosis and signal transduction [11]–[13]. Seven 14-3-3 isoforms (14-3-3β, 14-3-3γ, 14-3-3ε, 14-3-3ζ, 14-3-3η, 14-3-3σ, and 14-3-3τ) expressed in mammalian share about 50% amino acid identity and, consequently, highly similar protein conformations to form either homodimers or heterodimers that provide the functional basis for the target binding [14],[15]. Of particular interest is dimeric 14-3-3 proteins,which has a role in regulation of cell division. In HeLa cells, 14-3-3ζ cooperating with polo-like kinase 1 (PLK1) is required for mitotic exit and correct cytokinesis [16]. In cardiomyocyte, knockdown of 14-3-3ε causes decreased cardiac proliferation and a reduced number of cells in G2/M [17]. More recently it has been reported that14-3-3η localizes in the metaphase ‖ spindle of mouse eggs [18], and 14-3-3η is essential for normal meiotic spindle formation during in vitro maturation of mouse oocytes [19]. These results support the concept that 14-3-3 proteins are responsible for regulating mitosis and meiosis in mammalian cells.

Studies in HeLa cells have demonstrated that Ser323 is a primary 14-3-3 binding site in CDC25B, and this binding blocks the access of the catalytic site, thereby directly inhibiting the activity of CDC25B [20]. Ser323 phosphorylation is maintained into mitosis, but phosphorylation of Ser321 disrupts 14-3-3 binding to Ser323, mimicking the effect of inhibiting Ser323 phosphorylation on both CDC25B activity and localization [21]. In mouse oocytes, mutation Ser321 to Ala in CDC25B cannot bind to endogenous 14-3-3β whereas endogenous 14-3-3β can bind to wild type CDC25B [22]. Our previous studies in immunoprecipitation experiments demonstrated that 14-3-3ε interacts with phosphorylated CDC25B at Ser321 directly, but not with unphosphorylated CDC25B, and that the binding contributes to maintaining prophase ∣ arrest in the mouse oocyte [23]. However, there has been no proof of whether 14-3-3ε regulates the mitosis in fertilized mouse eggs. In the current study, we investigated whether 14-3-3ε binding with CDC25B-Ser321 which is phosphorylated by protein kinase A (PKA) regulates the early development of mouse embryos. We show here that 14-3-3ε interacts and cooperates with CDC25B phosphorylated at Ser321 regulating G2/M transition of mitotic progress of fertilized mouse eggs. We also show that knockdown of 14-3-3ε results in the block of G2/M transition and 14-3-3ε is primarily responsible for sequestering CDC25B in cytoplasm. Our studies suggest that 14-3-3ε binding to CDC25B-Ser321 phosphorylated by PKA induces mitotic arrest at one-cell stage by inactivation of MPF in fertilized mouse eggs.

## Results and discussion

### 14-3-3ε mRNA and protein expression in fertilized mouse eggs

**Figure 1:**
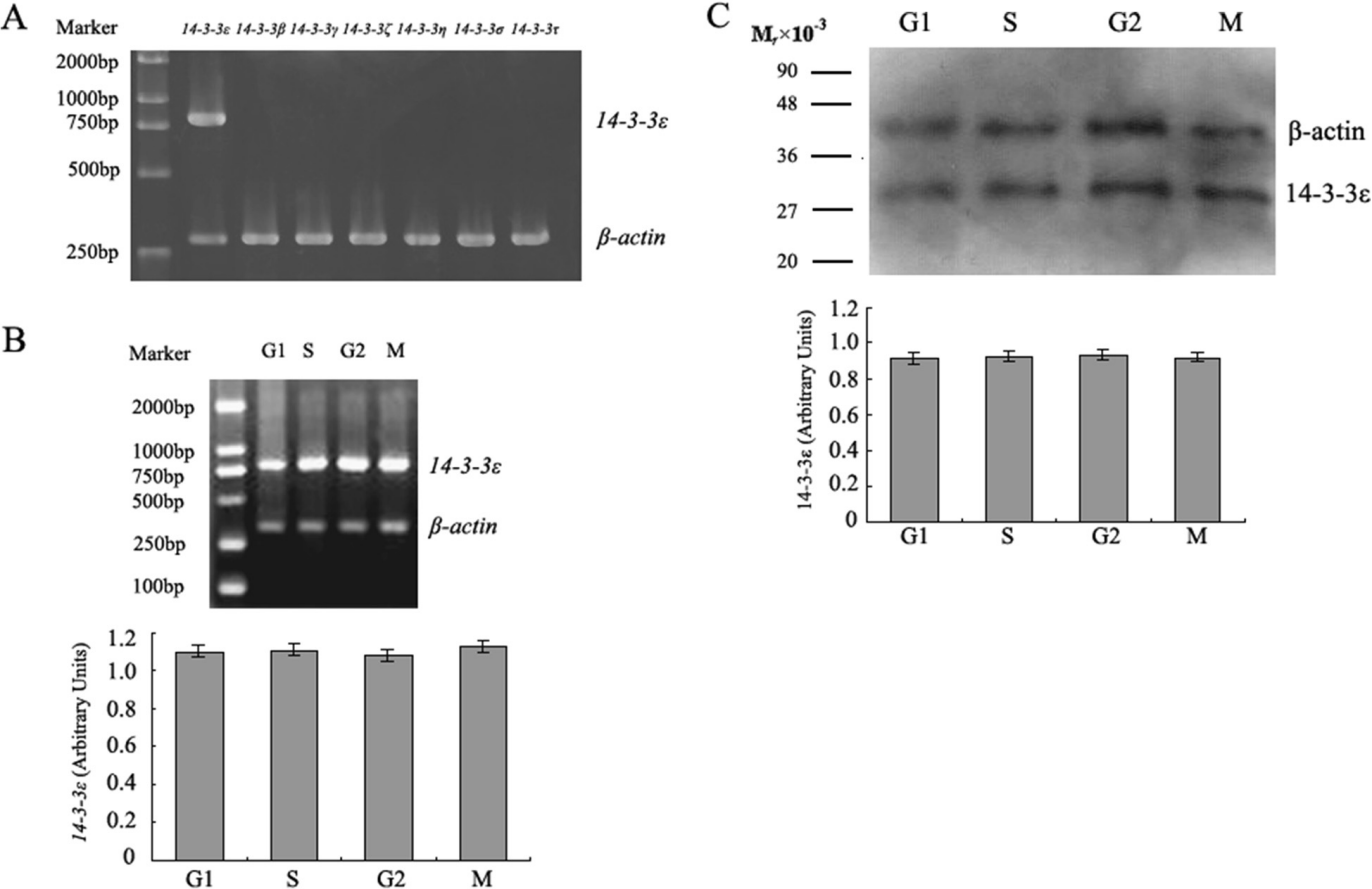
**The expression of 14-3-3ε in fertilized mouse eggs. A**: the mRNA expression levels of 14-3-3 isoforms at G1 phase of mouse fertilized eggs. mRNA of 150 fertilized mouse eggs was extracted in G1 phase. RT-PCR products using primers for 14-3-3 isoforms are observed in ethidium bromide-stained agarose gel. *14-3-3ε*, *14-3-3β*, *14-3-3γ*, *14-3-3ζ*, *14-3-3η*, *14-3-3σ*, and *14-3-3τ* represent seven 14-3-3 isoforms. **B**: the mRNA levels of *14-3-3ε* at G1, S, G2 and M phases (*P* >0.05). mRNA of 150 fertilized mouse eggs was extracted in G1, S, G2 and M phases, respectively. RT-PCR products using primers for *14-3-3ε* (800 bp) and *β–actin* (300 bp) are observed in ethidium bromide-stained agarose gel (upper panel). Densitometric quanitification represents the mRNA levels of *14-3-3ε* (lower panel) (*P* >0.05). **C:** Western blot analysis of 14-3-3ε protein expression (*P* >0.05). Immunoblots were performed for expression of 14-3-3ε (29 M_r_ × 10^−3^) and β-actin (43 M_r_ × 10^−3^) using anti-14-3-3ε or anti-β-actin antibodies (upper panel). Densitometric quanitification represents the protein expression of 14-3-3ε (lower panel) (*P* >0.05). 300 fertilized eggs are loaded onto each lane. Molecular weight of proteins (M_r_ × 10^−3^) is indicated. A *x*^2^ test was used to evaluate the differences of endogenous *14-3-3ε* mRNA expression levels or protein expression of 14-3-3ε between multiple experimental groups. Bars represent means ± S.D of three independent experiments.

We have previously demonstrated the only 14-3-3ε, one of the seven 14-3-3 isoforms, existed in GV and GVBD mouse oocytes and the expression of 14-3-3ε remained unchanged during GV and GVBD stages [23]. Fertilized mouse eggs at G1 phase were collected and used to amplify the mRNA of seven 14-3-3 isoforms. The results of RT-PCR showed that only *14-3-3ε* existed in G1 phase of fertilized mouse eggs (Figure 1A). In order to determine the expression levels of 14-3-3ε in fertilized mouse eggs, RT-PCR and Western blot were used to detect the mRNA and protein expression of 14-3-3ε, respectively, in G1, S, G2 and M phases. RT-PCR and Western blot analysis revealed that *14-3-3ε* mRNA expression and 14-3-3ε protein expression were present at constant levels at four phases of fertilized mouse eggs (*P* >0.05) (Figure 1B and C). Contrary to our results, Santanu De and his colleagues [18] have reported that mouse mature metaphase || -arrest eggs express all seven 14-3-3 isoforms and 14-3-3β, 14-3-3ε,14-3-3η and 14-3-3ζ appear in lesser amounts in mature metaphase || -arrest eggs than in immature oocytes.

### 14-3-3ε knockdown embryos failed in G2/M transition

**Figure 2:**
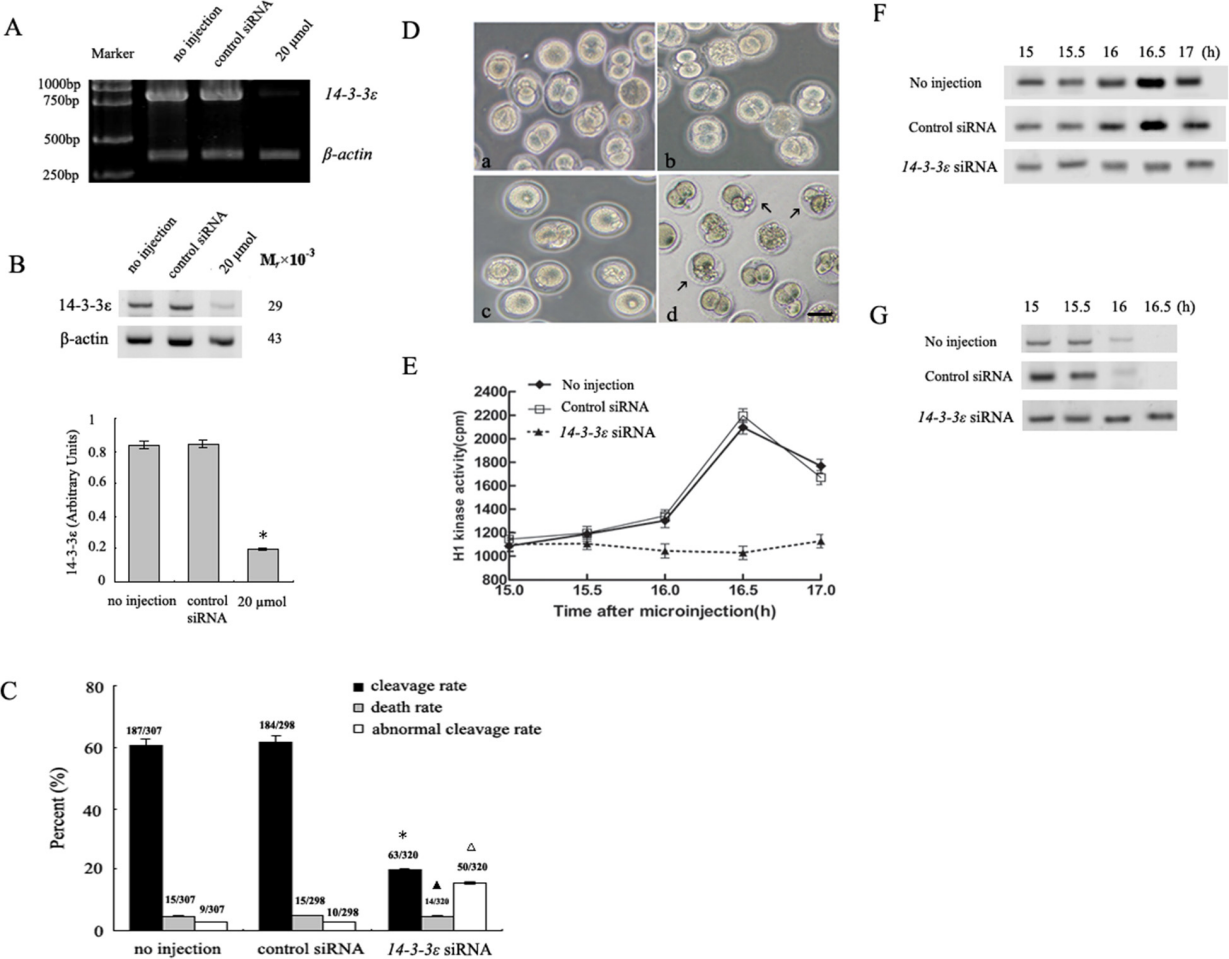
**Loss of 14-3-3ε results in block in G2/M transition. A**: 150 fertilized eggs microinjected with*14-3-3ε* siRNA or control siRNA (10 pl of 20 μmol) were collected 15 h after microinjection. RT-PCR is used for detection of mRNAs of *14-3-3ε* (800 bp) and *β-actin* (300 bp). **B**: Western blot analysis of 14-3-3ε protein expression at 15 h after microinjection of *14-3-3ε* siRNA using anti-14-3-3ε or anti-β-actin antibodies (upper panel). 300 fertilized eggs are loaded onto each lane. Densitometric quanitification represents the protein expression of 14-3-3ε (lower panel). A *x*^2^ test was used to evaluate the differences of endogenous 14-3-3ε expression between multiple experimental groups. Bars represent means ± S.D of three independent experiments. *P <0.01 vs. no injection or control siRNA group. **C**: The cleavage rate in cultured mouse embryos of *14-3-3ε* siRNA, control siRNA and no injections at 31 h after hCG injection. The cleavage rates were calculated with data from three independent experiments. The total number of eggs undergoing division, dead or abnormal cleavage is given on top of the bar graph. A *x*^2^ test was used to evaluate the differences between multiple experimental groups. Bars represent means ± S.D of three independent experiments. ^*^P <0.01, ^△^*P* <0.05, ^▲^*P* > 0.05 vs. no injection or control siRNA group. **D**: Morphology changes of mouse fertilized eggs in *14-3-3ε* siRNA microinjection and control groups. Representative images are shown. Bar =50 um. **a)** Eggs in no injection group. **b)** Eggs in control siRNA group. **c)** Eggs in *14-3-3ε siRNA* group. **d)** Abnormal cleavage in *14-3-3ε* siRNA group. The arrows indicate the eggs with abnormal cleavage. **E**, **F**: MPF activity was detected at indicated time points in *14-3-3ε* siRNA and control groups. For each point, eggs were lysed and MPF activity was examined by scintillation counting **(E)** and autoradiography **(F)**. One-way analysis of variance followed by a Least Significant Difference (LSD) test was used to evaluate the differences in the MPF activity assay. Bars represent means ± S.D of three independent experiments. **G**: Western blot analysis of phosphorylation status of CDC2-Tyr15. The eggs were collected at indicated time points after *14-3-3ε* siRNA injection in all the examined groups. 200 eggs are loaded onto each lane.

To explore the role of 14-3-3ε in G2/M transition of fertilized mouse eggs, a small interference RNA (*14-3-3ε* siRNA) at concentrations of 20 μmol (10 pl) was microinjected into the cytoplasm of fertilized mouse eggs at G1 stage (12 h after the hCG injection) to knock down endogenous *14-3-3ε*, which resulted in the strongest suppression without embryo lethality caused by over-microinjection. The fertilized eggs were then cultured in M16 medium at 37°C for 15 h to allow time for RNAi-mediated targeting of mRNA, which was assessed by RT-PCR (Figure 2A) and Western blotting (Figure 2B). Mouse fertilized eggs were either not microinjected or microinjected with control siRNA as control groups. As shown in Figure 2B, *14-3-3ε* siRNA microinjection caused 70–80% depletion of *14-3-3ε* (*P* <0.01 vs. no injection or control siRNA group). The morphology change and cleavage rate in each group were calculated after counting and observed under a phase-contrast microscope 19 h after the injection of siRNA (31 h after the hCG injection). In the two control groups, 60.9% (no injection) and 61.7% (injection of control siRNA) of embryos had reached the two-cell stage at 31 h after the hCG injection, and there was no significant difference between the two control groups (*P* > 0.05). A high number of embryos microinjected with *14-3-3ε* siRNA arrested at one-cell stage, and only 20% of embryos reached two-cell stage 19 h after the injection of siRNA (31 h after the hCG injection) (*P* <0.01 vs. no injection or control siRNA group). In addition, abnormal cleavage rate was significantly increased in the *14-3-3ε* siRNA eggs (*P* <0.05 vs. no injection or control siRNA group). Fewer than 5% of eggs were dead after the various injection (*P* > 0.05) (Figure 2C). The fertilized eggs injected control siRNA were morphologically normal compared to the no injection eggs (Figure 2D, a and b). Compared to the two controls, embryos of *14-3-3ε* knockdown group were 15% more likely to displayed abnormal cleavage (Figure 2D, c and d).

We have previously demonstrated the mitotic entry of mouse fertilized eggs is regulated by change in MPF activity [9]. In order to better understand whether *14-3-3ε* siRNA can inactivate MPF, we detected the MPF activity and phosphorylation status of CDC2-Tyr15. At 15 h after *14-3-3ε* siRNA microinjection, 5 fertilized eggs cultured in M16 medium were collected at indicated time points for the assay of MPF activity with histone H1 as the substrate. In control groups, MPF activity was consistently low at 15-15.5 h after control siRNA injection or no injection (27-27.5 h after the hCG injection), increased initially at 16 h (28 h after the hCG injection), and reached its maximal level at 16.5 h (28.5 h after the hCG injection) and began to decrease at 17 h (29 h after the hCG injection). In contrast, the MPF activity remained at low levels at 15-17 h after *14-3-3ε* siRNA injection (27-29 h after the hCG injection) (*P* <0.05 vs. no injection group or control siRNA injection group) (Figure 2E and F). Meanwhile, we measured the phosphorylation status of CDC2-Tyr15 in the control and *14-3-3ε* siRNA microinjection groups by Western blotting (Figure 2G). In control groups, there was strong inhibitory phosphorylation of CDC2-Tyr15 at 15-15.5 h, a reduced phosphorylation level at 16 h, and no signal at 16.5 h after control siRNA injection or no injection. In *14-3-3ε* siRNA injected eggs, the inhibitory phosphorylation of CDC2-Tyr15 was observed at 15-16.5 h after *14-3-3ε* siRNA injection. These results were consistent with the MPF activity measurements. These findings clearly indicate that *14-3-3ε* siRNA increases the phosphorylation of Tyr15 in CDC2 and the absence of 14-3-3ε blocks cell cycle progression by regulating the MPF activity at the G2/M transition of fertilized mouse eggs.

14-3-3 proteins play important roles in the regulation of cell development through binding to a large number of intracellular proteins containing specific phospho-serine/theonine motifs that are targeted by various classes of protein kinases. Meanwhile, 14-3-3 proteins as a critical integration point for many of the protein kinases and phosphatases that control the transition from G2 into M phase [24],[25]. One of the most well established roles for 14-3-3 proteins is in the control of cell cycle progression. In this study, we provide the experimental evidence for an important role of 14-3-3ε regulating mitotic progression. Cells lacking 14-3-3σ in marked contrast to normal cells, lead to impaired cytokinesis, loss of PLK1 at the midbody, and the accumulation of binucleate cells [26]. In HeLa cells, preventing phosphorylation of protein kinase Cε (PKCε) binding to 14-3-3 also causes defects in the completion of cytokinesis [27]. Similar to these studies, our studies showed that in the absence of 14-3-3ε, G2/M transition, as well as the cleavage rates, is impaired, and affected embryos display abnormal morphology, as indicated by irregular cleavage. Since downregulation of 14-3-3ε can interfere with the mitotic entry, it raises a particularly important question of whether or not 14-3-3ε can regulate MPF activation in fertilized mouse eggs. MPF inactivation obviously occurred in embryos injected with *14-3-3ε* siRNA by inhibitory phosphorylation of CDC2-Tyr15. Thus, depletion of 14-3-3ε protein after *14-3-3ε* RNAi treatment may prevent mitosis progression through inhibition of MPF activity.

### Co-injection of *14-3-3ε* mRNA and *Cdc25b*-Ser321A mRNA induces mitotic resumption in dbcAMP-arrested eggs

**Figure 3:**
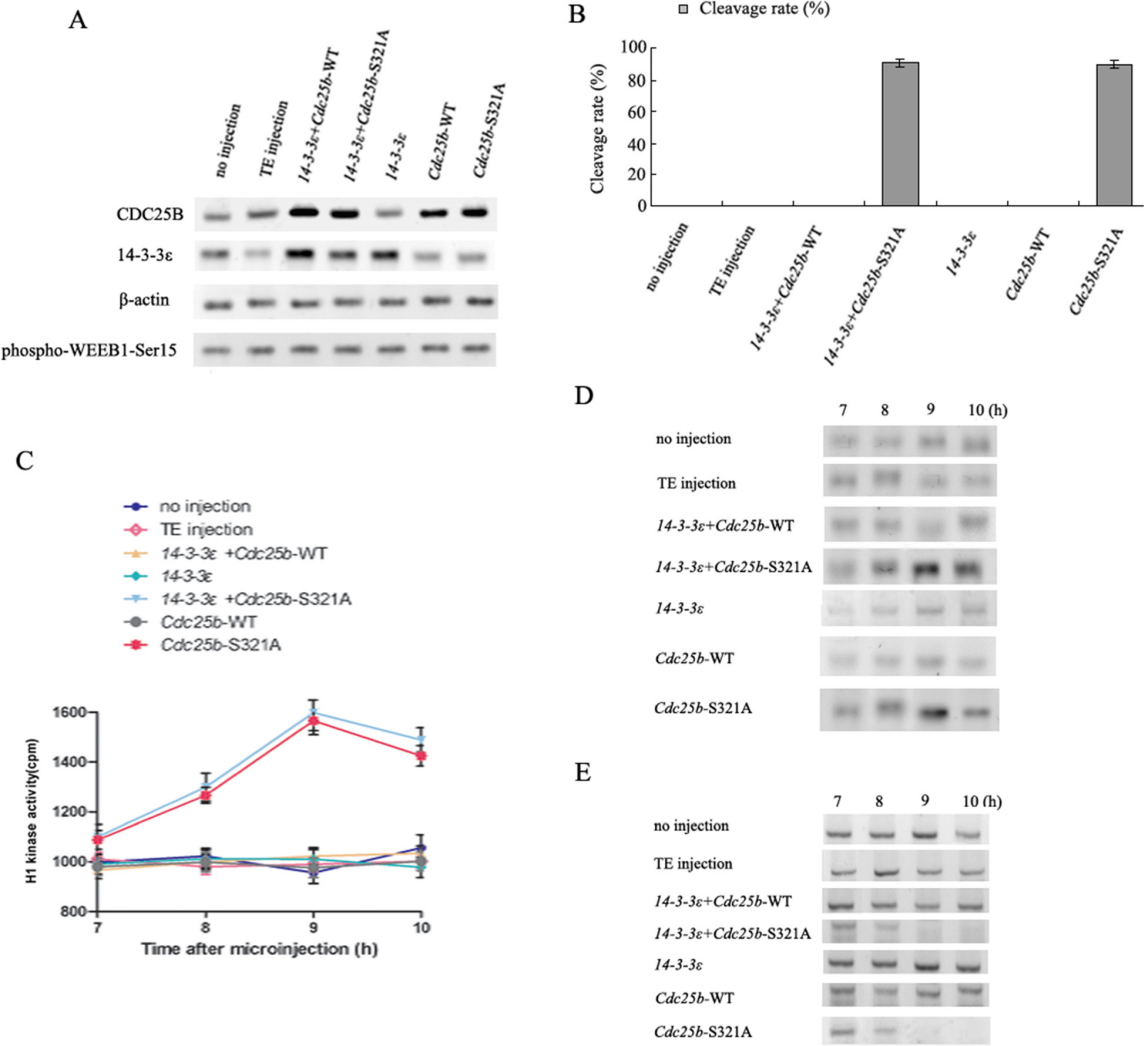
**Overexpression of 14-3-3ε and CDC25B mutants induces mitotic resumption. A**: Western blot analysis of CDC25B and 14-3-3ε expression at 7 h after various *14-3-3ε* mRNA and *Cdc25b* mRNA microinjection. Eggs were either not microinjected or microinjected with TE buffer as negative control groups and eggs injected with *Cdc25b*-S321A mRNA or *Cdc25b*-WT mRNA solely as the positive controls. Western blotting was performed using CDC25B, 14-3-3ε, phospho-WEE1B-Ser15 and β-actin antibodies in different microinjected groups. 200 eggs are loaded onto each lane. **B**: The cleavage rate in cultured mouse embryos 12 h after various *Cdc25b* mRNA and *14-3-3ε* mRNA injection in the presence of 2 mmol/l dbcAMP. A *x*^2^ test was used to evaluate the differences between multiple experimental groups. Bars represent means ± S.D of three independent experiments and 300 eggs are calculated for each group. **C**, **D**: MPF activity was detected at indicated time points in various mRNAs injection and control groups by scintillation counting **(C)** and autoradiography **(D)**. One-way analysis of variance followed by a Least Significant Difference (LSD) test was used to evaluate the differences in the MPF activity assay. Bars represent means ± S.D of three independent experiments. **E**: Western blot analysis of phosphorylation status of CDC2-Tyr15. 200 eggs are loaded onto each lane.

PKA activator, dibutyryl cAMP (dbcAMP) has a critical function in regulation of meiotic arrest and meiotic maturation in mouse oocytes [28],[29]. Our previous study demonstrated that 2 mmol/l membrane-permeable dbcAMP led to maximal G2 arrest, suggesting inhibition of the G2/M transition in fertilized mouse eggs [9]. Moreover, we previously demonstrated that 14-3-3ε binding to Ser321 of CDC25B blocked meiotic resumption in mouse oocytes [23]. To test whether 14-3-3ε binding to CDC25B-Ser321 affected the mitosis, mouse one-cell stage embryos (S phase, 21 h after the hCG injection) were first incubated in M16 medium containing 2 mmol/l dbcAMP and 1 h later microinjected with *14-3-3ε* mRNA solely or co-injected with mRNA of *Cdc25b*-S321A or *Cdc25b*-WT at a concentration of 300 μg/ml. Microinjection of *Cdc25b*-S321A mRNA or *Cdc25b*-WT mRNA solely served as the positive controls. Our recent study demonstrated WEE1B is a potential PKA target and Ser 15 phosphorylation of WEE1B is required for PKA-induced MPF inhibition in fertilized mouse eggs [30]. Since WEE1B may be phosphorylated by exogenous dbcAMP, we also detected the expression levels of various *Cdc25b* and *14-3-3ε* mRNAs in eggs with WEE1B-Ser 15 phosphorylation. Figure 3A showed that all the microinjected *Cdc25b* mRNAs and *14-3-3ε* mRNA were translated efficiently in mouse fertilized eggs under the condition that endogenous WEE1B-Ser 15 was phosphorylated by 2 mmol/l exogenous dbcAMP. In the negative control groups (no injection and TE injection groups), none of the mouse eggs was able to enter the M phase of mitosis because of inhibition of G2/M transition induced by dbcAMP which was similar to our previous results [9]. The cleavage rates in embryos co-injected with*14-3-3ε* mRNA and *Cdc25b*-Ser321A mRNA or injected with *Cdc25b*-Ser321A mRNA solely were significantly increased, nearly 90.9% of embryos had developed to the two-cell stage at 12 h after the microinjection (34 h after hCG injection) even WEE1B-Ser 15 was phosphorylated. However, none of the eggs co-injected with *14-3-3ε* mRNA and *Cdc25b*-WT mRNA or injected with *Cdc25b*-WT mRNA solely reached two-cell stage 12 h after the microinjection in the presence of dbcAMP, which was similar to the negative controls. In addition, eggs with injection of *14-3-3ε* mRNA alone still arrested at one-cell stage at 12 h after the microinjection, suggesting overexpression of 14-3-3ε had no effect on the mitotic entry with dbcAMP (Figure 3B).

We also measured the MPF activity and phosphorylation status of CDC2-Tyr15 in eggs injected with various mRNAs in the presence of dbcAMP. As anticipated, MPF activity in the eggs co-injected with *14-3-3ε* and *Cdc25b*-S321A mRNAs or injected with *Cdc25b*-Ser321A mRNA solely increasing initially at 8 h (30 h after hCG injection), and peaking at 9 h after microinjection (*P* <0.01 vs. two negative control groups, *14-3-3ε* mRNA injection group, *Cdc25b*-WT mRNA injection group or *14-3-3ε* and *Cdc25b*-WT mRNA co-injection group). In contrast, MPF activity remained at a relatively low level in the *14-3-3ε* mRNA or *Cdc25b*-WT mRNA solely injected, co-injection of *14-3-3ε* and *Cdc25b*-WT mRNA or two negative controls at 7-10 h after microinjection (29-32 h after hCG injection) (Figure 3C and D). Simultaneously, we detected the phosphorylation status of CDC2-Tyr15 in all of the examined groups (Figure 3E). In control groups, inhibitory phosphorylation of CDC2-Tyr15 was observed at 7-10 h after microinjection. Similar results were observed in *14-3-3ε* mRNA or *Cdc25b*-WT mRNA solely injected and co-injection of *14-3-3ε* and *Cdc25b*-WT mRNA groups, indicating that overexpression of neither 14-3-3ε solely nor 14-3-3ε and CDC25B-WT can dephosphorylate CDC2-Tyr15 in the presence of dbcAMP. On the contrary, strong CDC2-Tyr15 phosphorylation was found only at 7 h, and no phosphorylation signal at 9 h co-injected with *14-3-3ε* and *Cdc25b*-Ser321A mRNAs or injected with *Cdc25b*-Ser321A mRNA solely. These results were consistent with the MPF activity measurements. These data suggest that 14-3-3ε binding to CDC25B-Ser321 phosphorylated by PKA induces mitotic arrest at one-cell stage by inactivation of MPF.

CDC25B is a key regulator of entry into mitosis, and its activity and localization are regulated by binding of the 14-3-3 proteins [20]. Our previous studies demonstrated that the Ser321 of CDC25B plays a critical regulatory role in the G2/M transition by activating MPF in fertilized mouse eggs. Overexpression of CDC25B-Ser321A in fertilized mouse eggs can induce CDC2-Tyr15 dephosphorylation and overcome G2 arrest induced by dbcAMP, whereas wild type CDC25B has no effect on mitotic resumption [9]. In the present study, the mitotic arrest in the fertilized mouse eggs induced by dbcAMP was completely reversed by co-expression of 14-3-3ε and CDC25B-Ser321A despite of phosphorylation of endogenous WEE1B-Ser 15, which was similar to the injection of CDC25B-Ser321A solely. In contrast, none of the eggs co-expression of 14-3-3ε and CDC25B-WT or expression of CDC25B-WT solely could efficiently override the G2 arrest in the presence of dbcAMP. In addition, overexpression of 14-3-3ε alone did not affect the division. These findings strongly suggest that Ser321 of CDC25B is the major site for 14-3-3ε binding and this binding likely blocks access to MPF, required for mitotic entry.

### Co-localization of endogenous 14-3-3ε and CDC25B in fertilized eggs

**Figure 4:**
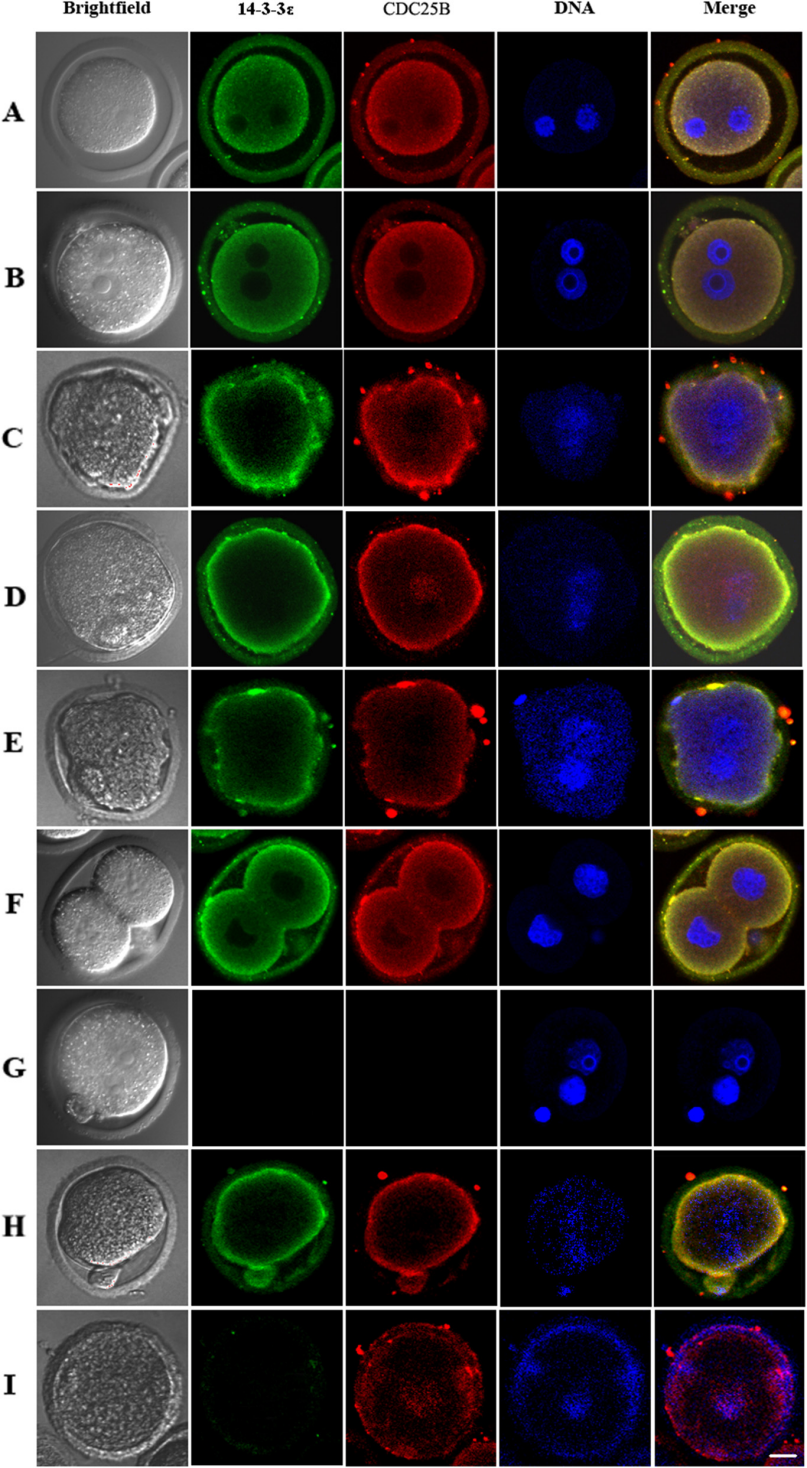
**Co-localization of endogenous 14-3-3ε and CDC25B in fertilized eggs.** The representative cells were fixed, permeabilized and immunolabeled for confocal microscopy at different phases. **A**, **B**: The CDC25B signals and 14-3-3ε signals were co-localized primarily in the cytoplasm at G1and S phases, respectively. **C**: The CDC25B signals and 14-3-3ε signals were observed in the cytoplasm at early G2 phase. **D**: The 14-3-3ε signals were detected in the cytoplasm, but nuclear accumulation of CDC25B signals were observed at late G2 phase. **E**, **F**: The CDC25B signals and 14-3-3ε signals distributed in the cytoplasm again at early M phase and late M phase, respectively. **G**: Negative control showed no signal of CDC25B and 14-3-3ε. **H**: CDC25B was localized in the cytoplasm 15 h after control siRNA microinjection at early G2 phase. **I**: The CDC25B signals moved to the nucleus from cytoplasm 15 h after *14-3-3ε* siRNA injection at early G2 phase. Bar =20 um.

Several previous studies demonstrated that binding of 14-3-3 to CDC25B induce the redistribution of CDC25B from the nucleus to the cytoplasm [20],[31]. Therefore, we observed the co-localization of endogenous CDC25B and 14-3-3ε at every phase of cell cycle in fertilized mouse eggs with indirect immunofluorescence. We examined 30 different eggs from G1, S, early G2, late G2, early M and late M phases, respectively, and all showed the same pattern of immunofluorescent staining. As shown in Figure 4, red fluorescent CDC25B signals and green fluorescent 14-3-3ε signals were co-localized primarily in the cytoplasm at G1 and S phases, respectively (Figure 4A and B). In early G2 phase eggs, 14-3-3ε and CDC25B signals were observed in the cytoplasm (Figure 4C). Partial CDC25B signals translocated to the nucleus of eggs, whereas 14-3-3ε signals still remained in the cytoplasm at the late G2 phase (Figure 4D). However, the CDC25B signals and 14-3-3ε signals in the nucleus apparently weakened and became distributed in the cytoplasm again at early and late M phases, respectively (Figure 4E and F). Negative control showed no signal of CDC25B and 14-3-3ε (Figure 4G).

To further understand whether 14-3-3ε knockdown can affect the distribution of CDC25B, we determined the localization of endogenous CDC25B and 14-3-3ε 15 h (27 h after the hCG injection, G2 phase) after *14-3-3ε* siRNA or control siRNA microinjection by indirect immunofluorescence. The red fluorescent CDC25B signals were localized in the cytoplasm at early G2 phase in control siRNA injection group in 27 of 30 eggs which was similar to the normal eggs of early G2 phase in Figure 4C (Figure 4H), while CDC25B signals were highly concentrated on the nucleus in 25 of 30 eggs, indicating CDC25B transfers from cytoplasm to the nucleus at early G2 phase when 14-3-3ε knocked down (Figure 4I). None of the fertilized eggs injected with *14-3-3ε* siRNA showed the 14-3-3ε staining (Figure 4I) compared to the control siRNA injection eggs (Figure 4H). These data suggest that 14-3-3ε may control the cytoplasmic localization of CDC25B.

It has been shown that endogenous CDC25B is mainly nuclear, but a fraction resides in the cytoplasm during the G2 phase of the cell cycle in HeLa cells [32]. Contrary to this study, our immunofluoresence experiments revealed a restricted cytoplasmic co-localization of 14-3-3ε and CDC25B at G1, S, early G2 and M phases in fertilized eggs. Moreover, we also observed that CDC25B transferred from cytoplasm to the nucleus at the late G2 phase, together with previous studies [31],[33], support that CDC25B can actively shuttle in and out of the nucleus of the fertilized eggs at G2 phase whereas 14-3-3ε may bind to CDC25B to sequester CDC25B in the cytoplasm. Additionally, our observation that the cytoplasmic localization of CDC25B was altered at early G2 phase following deletion of 14-3-3ε suggests that 14-3-3ε might directly modulate CDC25B distribution.

### Co-localization of exogenously expressed 14-3-3ε and CDC25B

**Figure 5:**
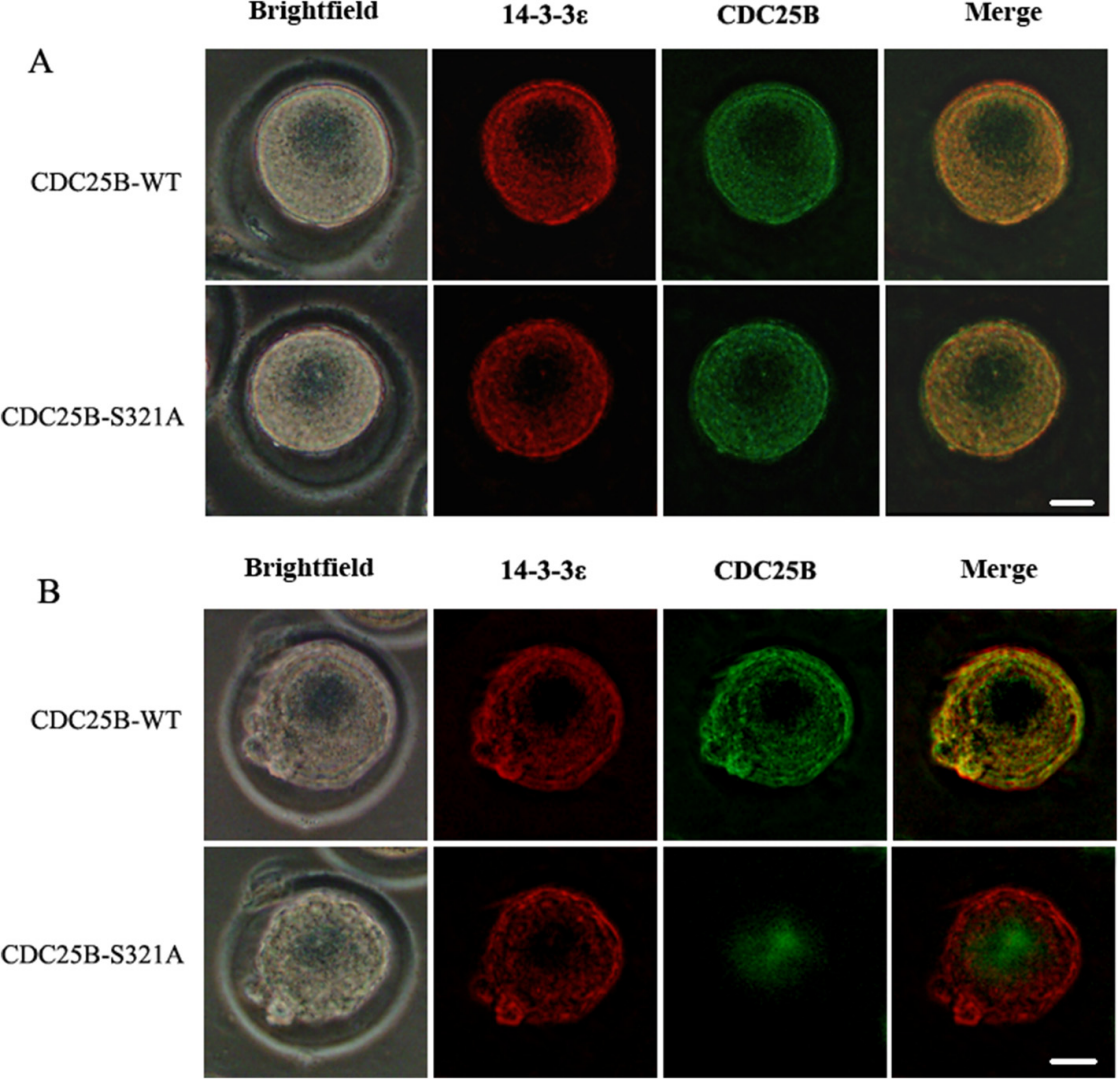
**Co-localization of exogenously expressed 14-3-3ε and CDC25B. A**: GFP-CDC25B and RFP-14-3-3ε were co-localized primarily in the cytoplasm at early G2 phase in two groups (25/30 and 26/30 eggs, respectively). **B**: GFP-CDC25B-S321A translocated to the nucleus (24/30 eggs) whereas GFP-CDC25B-WT were detected in the cytoplasm co-localized with 14-3-3ε (25/30 eggs) at late G2 phase in fertilized mouse egg. 14-3-3ε localized predominantly in the cytoplasm at late G2 phase in CDC25B-S321A group. Bar =20 um.

To confirm the subcellular localization of exogenous 14-3-3ε and CDC25B, the pEGFP-CDC25B-WT and pEGFP-CDC25B-S321A plasmids were co-injected with pRFP-HA-14-3-3ε into fertilized mouse eggs, respectively, at the G1 phase (19 h after hCG injection), and then the microinjected eggs were transferred into M16 medium containing 2 mmol/l dbcAMP. Thirty eggs from early G2 and late G2 phases for each group were analyzed, respectively. As shown in Figure 5A, when mouse embryos injected with pEGFP-CDC25B-WT/pRFP-HA-14-3-3ε or pEGFP-CDC25B-S321A/pRFP-HA-14-3-3ε entered early G2 phase, green fluorescent CDC25B signals and red fluorescent 14-3-3ε signals were co-localized primarily in the cytoplasm of mouse fertilized egg. Then, the green fluorescent signals of CDC25B-S321A were translocated to the nucleus whereas CDC25B-WT signals were observed in the cytoplasm of mouse fertilized egg without nucleus accumulation at late G2 phase. The red fluorescent 14-3-3ε signals were detected primarily in the cytoplasm in both CDC25B-WT and CDC25B-S321A groups at late G2 phases (Figure 5B). These data suggest that CDC25B cannot transfer to the nucleus when CDC25B-Ser321 is phosphorylated and cytoplasmic retention of CDC25B-S321A at early G2 phase is required for activating MPF.

It has been reported that 14-3-3β and 14-3-3ε specifically bind to Ser309 of CDC25B and that mutation of CDC25B Ser309 to Ala impairs 14-3-3 binding and completely abolished the cytoplasmic localization of CDC25B [34]. In contrast, we observed a cytoplamic localization of CDC25B-S321A at early G2 phase and then CDC25B-S321A transferred from cytoplasm to nucleus at late G2 phase. These results are consistent with the observations that human CDC25C in which the mutation of Ser216 to Ala at the 14-3-3 binding site does not completely abolish its cytoplasmic localization [35]. An intrinsic nuclear localization sequence (NLS) and a nuclear export sequence (NES) lies between the N-terminal regulatory domain and the C-terminal catalytic domain of CDC25C [35],[36]. The major 14-3-3 binding sites of human CDC25C-Ser216 and Xenopus CDC25C-Ser287 are located right next to the NLS [35],[37]. Mutation of the nuclear export sequence makes CDC25B less efficient in inducing mitosis in the cytoplasm [32]. Moreover, our previous results showed that deletion of functional nuclear export sequence in the N-terminus of CDC25B is sufficient to abrogate CDC25B export in mouse oocytes. Interference with nuclear export reduced the ability of CDC25B protein to induce GVBD suggesting that CDC25B is needed to activate CDC2/CyclinB in the cytoplasm (unpublished data, Yu B). The Ser321 of mouse CDC25B, which corresponds to the Ser323 of human CDC25B and the Ser287 of Xenopus CDC25C, when mutated to a nonphosphorylatable alanine, is incapable of affecting the NLS and NES, thus accelerating mitosis compared to the CDC25B-WT at the presence of dbcAMP in our present study. Although it is unclear what causes the cytoplasmic localization of CDC25B-S321A without 14-3-3ε binding at early G2 phase, the nuclear export of CDC25B-S321A is likely regulated by NES. In HeLa cells, CDC25C was not exclusively localized to the nucleus unless both 14-3-3 binding and NES function were disrupted [35]. Thus, CDC25B-S321A may have a normal NES and the accumulation of CDC25B-S321A in nucleus at early G2 phase may also require inactivation of its NES.

An important observation made by Kornbluth and colleagues is that 14-3-3 binding to Xenopus CDC25 phosphorylated on S287 protects this mitotic phosphatase from premature dephosphorylation and activation [38]. Removal of 14-3-3 proteins binding to phosphorylated CDC25 during interphase is one of the early steps in mitotic activation during the pathway of DNA-responsive checkpoints [39]. Several reports demonstrate that the complex of CDC2/CyclinB1 is first activated on centrosomes and full activation occurs in the nucleus [40],[41]. Moreover, cytoplasmic CDC25B may mediate the activation of centrosomal CDK1 (CDC2) in late prophase [42]. Thus, it is possible that CDC25B-S321A activates the MPF much more efficiently in cytoplasm and then makes a full activation of MPF in nucleus whereas phosphorylated Ser321 on CDC25B-WT binding to 14-3-3ε fails to dephosphorylate activating MPF under conditions that maintains exogenous dibutyryl cAMP.

Our previous studies have demonstrated that the CDC25B-S321 is phosphorylated at the G1 and S phases in the fertilized mouse eggs, whereas no phosphorylation of CDC25B-S321 was observed at the G2 and M phases in vivo, suggesting that unphosphorylatable CDC25B is required for activating MPF [9],[10]. In xenopus eggs, protein phosphatase 1 (PP1) is required for dephosphorylation of CDC25 at Ser287 for initiation of mitosis [37]. Based on our findings, we propose a model that phosphorylation of CDC25B-Ser321 by PKA allows 14-3-3ε to bind CDC25B, which results in CDC25B being sequestered in the cytoplasm at G1 and S phases, whereas phosphorylated CDC25B is dephosphorylated by protein phosphatase, activated under appropriate conditions without 14-3-3ε binding at early G2 phase, and stimulates cytoplasmic MPF initially at early G2 phase and then nucleus MPF at late G2 phase, triggering G2/M transition in fertilized mouse eggs. Furthermore, downregulation of 14-3-3ε inhibiting MPF activity may due to the translocation of CDC25B to the nucleus when 14-3-3ε knocked down, which could not activate the MPF efficiently in the cytoplasm at early G2 phase. Additional regulatory mechanisms of 14-3-3ε for the suppression of the G2/M phase when 14-3-3ε deleted cannot be ruled out in the mitosis of fertilized mouse eggs. 14-3-3ε deletion leads to significant accumulation of cardiomyocytes in the G0/G1phase by upregulation of p27^Kip1^ and downregulation of Cyclin E1, respectively, which in turn is likely to depress progression into G2/M [17]. Proteomic analysis of interphase and mitotic HeLa cells have demonstrated that several known 14-3-3 targets bound to 14-3-3 proteins, including the cell cycle regulator WEE1, the Par-1a (C-TAK1) and Par-1b (EMK) kinases, β-tubulin, which have been implicated in regulating cell polarity, microtubule dynamics, and the cell division cycle [43],[44]. Thus, it is likely that these factors, such as cyclin E1, WEE1 or β-tubulin may contribute to the cell cycle defects in 14-3-3ε knockdown fertilized mouse eggs.

In this study, we did not give the evidence that which protein phosphates dephosphorylate and activate CDC25B at early G2 phase. In the future, it is of great importance to probe the molecular mechanisms how CDC25B is dephosphorylated and activated at early G2 phase under appropriate conditions in fertilized mouse eggs. Moreover, additional functional experiments will be needed to determine the timing of 14-3-3ε absence from CDC25B in the fertilized mouse eggs.

## Conclusions

In this study, for the first time, we provide the experimental evidence for an important role of 14-3-3ε regulating G2/M transition and CDC25B distribution in the fertilized mouse eggs. The results of the study indicate that 14-3-3ε is required for the mitotic entry in the fertilized mouse eggs. The activation of MPF by CDC25B in the cytoplasm at early G2 phase is responsible for initiation of G2/M transition in the fertilized mouse eggs. These results suggest that 14-3-3ε binding to CDC25B-S321 phosphorylated by PKA blocks the mitotic division by inactivation of MPF at G1 and S phases in fertilized mouse eggs. 14-3-3ε knockdown also resulting in the mitotic arrest may due to the lost of cytoplasmic localization of CDC25B, thereby inactivating MPF initially in the cytoplasm.

## Methods

### Animals and reagents

Kunming strain mice (females of 4 weeks age, 18-20 g weight and males of 8 weeks age, 30-35 g weight) were obtained from the Department of Laboratory Animals, China Medical University (CMU). All experiments were performed at CMU in accordance with the National Institutes of Health guidelines for the Care and Use of Laboratory Animals. The protocol for animal handling and the treatment procedures were reviewed and approved by the CMU Animal Care and Use Committee. Reagents, unless otherwise specified, were from Sigma.

### Collection and culture of mouse embryos

One-cell stage mouse embryos were collected and cultured according to the method described by our previous report [9] on the basis of the method of Hogan and Constantini [45]. The embryos were incubated in M16 medium under equilibrated mineral oil at 37°C, 5% CO_2_ in air after injection with various siRNA or mRNA or plasmids.

### Microinjection and morphology analysis

Various CDC25B or 14-3-3ε plasmids and *14-3-3ε* or *Cdc25b* mRNAs were microinjected into the nucleus or cytoplasm of one-cell embryos at G1 or S phases according to our previous report [9],[10]. Typical injection volume was 5% (10 pl, cytoplasm) and 1% (2 pl, nuclear) of total cell volume per egg. Messenger RNA was diluted to various concentrations in TE buffer (pH 7.4), respectively, without nuclease contaminant. Eggs in control groups were either not microinjected or microinjected with TE buffer.

Mitotic stages (G1, S, G2 and M phases) were defined as previously described. G1 phase: 10-19 h after hCG injection. S phase: 19-25 h after hCG injection. G2 phase: 25-28 h after hCG injection. M phase: 28-31 h after hCG injection [46]. The rate of cleavage, namely the number of two-cell embryos resulted from one-cell embryos division was counted from three independence experiments under a phase-contrast microscope 31 h or 34 h after the hCG injection in the absence or presence of dbcAMP in control and microinjection groups. Morphological analysis was performed by inverted microscope.

### Microinjection of siRNA in mouse embryos

For targeted knockdown of *14-3-3ε*, the siRNA for mouse *14-3-3ε* was microinjected into the cytoplasm of one-cell embryos at G1 phase. The G1 phase embryos were transferred to M2 medium, and the cytoplasm was injected with 10 pl of siRNA (20 μmol) solution. Following microinjection, embryos were transferred to M16 medium and incubated at 37°C in 5% CO_2_ for 15 h, and the rate of cleavage was scored with a DMI4000B inverted microscope fitted with a differential interference contrast (DIC) lens. The sequence of *14-3-3ε* siRNA was 5′-UGUACAUCCAGAAUGUCACAACAGA-3′. A 25 bp duplex oligoribonucleotide of RNAi Neg Ctl LO GC (Invitrogen) was used as the control siRNA. All siRNA duplexes were resuspended in 1 ml DEPC-treated water according to the manufacturer’s instructions and stored in single use aliquots at -20°C.

### RT-PCR

mRNAs were extracted from fertilized mouse eggs in the G1,S,G2 and M phases using the EllustraTM QuickPrep MicromRNA Purification Kit (GE Healthcare UK Limited,UK). RT-PCR was performed using RNA PCR Kit (AMV) Ver 3.0 (TakaRa). The *14-3-3ε* primer was designed according to published mouse cDNA. *14-3-3ε* (NM_009536.4), forward primer: 5′-TATCTCGAGGCCGCAATGGATGATCGGGAG -3′ and reverse primer: 5′-CGCGGATCCTACGTCTCACTGATTCTCATC-3′. *β-actin* (NM_007393.3), forward primer: 5′-GTGGCATCCATGAAACTACAT-3′ and reverse primer: 5′-AACGCAGCTCAGTAACAGTC-3′. The primers of *14-3-3β, 14-3-3γ, 14-3-3ζ, 14-3-3η, 14-3-3σ,* and *14-3-3τ* were designed as previously described [23]. The RT reaction was carried out for one cycle at 50°C for 30 min; 99°C for 5 min; 5°C for 5 min. Aliquots of 5 ml of first-strand cDNA were mixed with 20 ml of the PCR mixture. The PCR reaction was carried out in three steps as follows: 94°C for 2 min (one cycle); 94°C for 30 sec, 58°C for 30 sec, and 72°C for 1 min (35 cycle); 72°C for 10 min (one cycle). The PCR products were analyzed by electrophoresis on 1.2% agarose gel stained with ethidium bromide to visualize PCR products on a UV transilluminator. Densitometry of bands was performed with Quantity One Software, densitometry of *14-3-3ε* bands/densitometry of *β-actin* bands was used as quantitation of mRNA expression of *14-3-3ε*.

### Assay of MPF activity

MPF kinase activity was measured using histone H1 kinase assay [47]. Five fertilized eggs cultured in M16 medium were collected, washed in collection buffer (PBS containing 1 mg/ml polyvinyl alcohol, 5 mM EDTA, 10 mM Na_3_VO_4_, and 10 mM NaF), and then transferred to an Eppendorf tube containing 5 μl collection buffer. The Eppendorf tube was immediately stored at -70°C until the kinase assay was performed. The kinase assay was performed according to a similar procedure to that described in our previous report [9],[10].

### Western blot

Protein extracts of mouse fertilized eggs were prepared by adding approximately 200 or 300 eggs in a minimal volume of collection medium to 20 μl of protein extraction buffer (100 mM NaCl, 20 mM Tris-HCL [pH 7.5], 0.5% Triton X-100, 0.5% NP-40) containing 1 mM phenylmethylsulfonyl fluoride and 1 μg/ml leupeptin and pepstatin. Laemmli sample buffer was added to the protein extracts, and the mixture was boiled for 5 min and subjected to a 10% SDS-PAGE gel. For immunoblotting, the fractionated proteins were transferred to a nitrocellulose membrane. The membrane was blocked with 3% BSA in Tris-buffered saline containing 0.05% Tween 20 and probed with primary antibody against 14-3-3ε (1:200), CDC25B(1:200), β-actin (1:400) (Santa Cruz Biotechnology) and phospho-WEE1B-Ser15 antibody (1:100) (Signalway Antibody Co., Ltd.) overnight at 4°C. The membrane was then incubated with a horseradish peroxidase-conjugated anti-mouse, anti-goat or anti-rabbit secondary antibody at 1:3000 (Beijing Zhongshan Biotechnology). The proteins were detected by using an enhanced chemiluminescence detection system (Piece Biotechnology).

The proteins expression of 14-3-3ε and β-actin were detected by Western blot. Densitometry of bands was performed with Quantity One Software, densitometry of 14-3-3ε bands/densitometry of β-actin bands was used as quantitation of endogenous 14-3-3ε expression.

### Immunofluorescence

Embryos were washed in PBS with 0.1% BSA, and fixed in 4% paraformaldehyde in PBS (pH7.4) for 1 h at room temperature. After being permeablilzed with 0.1% TritonX-100 in PBS at room temperature for 30 min, embryos were blocked in 5% BSA in PBS for 1 h and incubated overnight at 4°C with monoclonal mouse anti-14-3-3ε antibody (1:100, Santa Cruz Biotechnology) and polyclonal goat anti-CDC25B antibody (1:100, Santa Cruz Biotechnology) at 4°C . After being washed three times in PBS with 0.1% BSA, the embryos were incubated with FITC-conjugated goat anti-mouse secondary antibody (1:100, Beijing Boaoshen Biotechnology) and TRITC-conjugated rabbit anti-goat secondary antibody (1:100, Beijing Zhongshan Biotechnology) at 37°C for 1 h. Then, The DNA was stained with 25 μg/ml Hoechst33258 for 10 min at room temperature. The signals of subcellular localization of endogenous 14-3-3ε, CDC25B and DNA stained were detected by a Laser Confocal Scanning Microscope at 488 nm, 530 nm and 260 nm, respectively.

### Statistical analysis

All experiments were performed independently at least three times. A *x*^2^ test or one-way analysis of variance followed by a Least Significant Difference (LSD) test was used to evaluate the differences between multiple experimental groups with SSPS software (version 13.0). A probability level of 0.05 was considered significant.

## Competing interests

The authors have no competing interests.

## Authors’ contributions

CC, BY and EW designed the project and gave final approval of the version to be published. XR, XD and XQ performed the experiments. CC and DL analyzed the data and combined to draft the manuscript. XZ contributed reagents/materials/analysis tools. All authors read and approved the final manuscript.

## References

[B1] McGowan CH Russell P Human Wee1 kinase inhibits cell division by phosphorylating p34cdc2 exclusively on Tyr15 EMBO J 1993 12 75 85 8428596 10.1002/j.1460-2075.1993.tb05633.x PMC413177

[B2] Mueller PR Coleman TR Kumagai A Dunphy WG Myt1: a membrane-associated inhibitory kinase that phosphorylates Cdc2 on both threonine-14 and tyrosine-15 Science 1995 270 86 90 7569953 10.1126/science.270.5233.86

[B3] Parker LL Piwnica-Worms H Inactivation of the p34cdc2-cyclin B complex by the human WEE1 tyrosine kinase Science 1992 257 1955 1957 1384126 10.1126/science.1384126

[B4] Morgan DO Principles of CDK regulation Nature 1995 374 131 134 7877684 10.1038/374131a0

[B5] Strausfeld U Labbé JC Fesquet D Cavadore JC Picard A Sadhu K Russell P Dorée M Dephosphorylation and activation of a p34cdc2/cyclin B complex in vitro by human CDC25 protein Nature 1991 351 242 245 1828290 10.1038/351242a0

[B6] Donzelli M Draetta GF Regulating mammalian checkpoints through Cdc25 inactivation EMBO Rep 2003 4 671 677 12835754 10.1038/sj.embor.embor887 PMC1326326

[B7] Trinkle-Mulcahy L Lamond AI Mitotic phosphatases: no longer silent partners Curr Opin Cell Biol 2006 18 623 631 17030123 10.1016/j.ceb.2006.09.001

[B8] Nishijima H Nishitani H Seki T Nishimoto T A dual-specificity phosphatase Cdc25B is an unstable protein and triggers p34 (cdc2)/cyclin B activation in hamster BHK21 cells arrested with hydroxyurea J Cell Biol 1997 138 1105 1116 9281587 10.1083/jcb.138.5.1105 PMC2136770

[B9] Cui C Zhao H Zhang Z Zong Z Feng C Zhang Y Deng X Xu X Yu B CDC25B acts as a potential target of PRKACA in fertilized mouse eggs Biol Reprod 2008 79 991 998 18633139 10.1095/biolreprod.108.068205

[B10] Xiao J Liu C Hou J Cui C Wu D Fan H Sun X Meng J Yang F Wang E Yu B Ser149 is another potential PKA phosphorylation target of Cdc25B in G2/M transition of fertilized mouse eggs J Biol Chem 2011 286 10356 10366 21212267 10.1074/jbc.M110.150524 PMC3060489

[B11] Jin J Smith FD Stark C Wells CD Fawcett JP Kulkarni S Metalnikov P O'Donnell P Taylor P Taylor L Zougman A Woodgett JR Langeberg LK Scott JD Pawson T Proteomic, functional, and domain-based analysis of in vivo 14-3-3 binding proteins involved in cytoskeletal regulation and cellular organization Curr Biol 2004 14 1436 1450 15324660 10.1016/j.cub.2004.07.051

[B12] Mackintosh C Dynamic interactions between 14-3-3 proteins and phosphoproteins regulate diverse cellular processes Biochem J 2004 381 329 342 15167810 10.1042/BJ20031332 PMC1133837

[B13] Dougherty MK Morrison DK Unlocking the code of 14-3-3 J Cell Sci 2004 117 1875 1884 15090593 10.1242/jcs.01171

[B14] Aitken A 14-3-3 proteins: a historic overview Semin Cancer Biol 2006 16 162 172 16678438 10.1016/j.semcancer.2006.03.005

[B15] Chaudhri M Scarabel M Aitken A Mammalian and yeast 14-3-3 isoforms form distinct patterns of dimers in vivo Biochem Biophys Res Commun 2003 300 679 685 12507503 10.1016/s0006-291x(02)02902-9

[B16] Du J Chen L Luo X Shen Y Dou Z Shen J Cheng L Chen Y Li C Wang H Yao X 14-3-3zeta cooperates with phosphorylated Plk1 and is required for correct cytokinesis Front Biosci (Schol Ed) 2012 4 639 650 22202082 10.2741/s290

[B17] Kosaka Y Cieslik KA Li L Lezin G Maguire CT Saijoh Y Toyo-oka K Gambello MJ Vatta M Wynshaw-Boris A Baldini A Yost HJ Brunelli L 14-3-3ε plays a role in cardiac ventricular compaction by regulating the cardiomyocyte cell cycle Mol Cell Biol 2012 32 5089 5102 23071090 10.1128/MCB.00829-12 PMC3510533

[B18] De S Marcinkiewicz JL Vijayaraghavan S Kline D Expression of 14-3-3 protein isoforms in mouse oocytes, eggs and ovarian follicular development BMC Res Notes 2012 5 57 doi:10.1186/1756-0500-5-57 22264317 10.1186/1756-0500-5-57 PMC3292963

[B19] De S Kline D Evidence for the requirement of 14-3-3eta (YWHAH) in meiotic spindle assembly during mouse oocyte maturation BMC Dev Biol 2013 13 10 doi:10.1186/1471-213X-13-10 23547714 10.1186/1471-213X-13-10 PMC3620909

[B20] Forrest A Gabrielli B Cdc25B activity is regulated by 14-3-3 Oncogene 2001 20 4393 4401 11466620 10.1038/sj.onc.1204574

[B21] Astuti P Boutros R Ducommun B Gabrielli B Mitotic phosphorylation of Cdc25B Ser321 disrupts 14-3-3 binding to the high affinity Ser323 site J Biol Chem 2010 285 34364 34370 20801879 10.1074/jbc.M110.138412 PMC2966050

[B22] Pirino G Wescott MP Donovan PJ Protein kinase A regulates resumption of meiosis by phosphorylation of Cdc25B in mammalian oocytes Cell Cycle 2009 8 665 670 19223768 10.4161/cc.8.4.7846

[B23] Meng J Cui C Liu Y Jin M Wu D Liu C Wang E Yu B The role of 14-3-3ε interaction with phosphorylated Cdc25B at its Ser321 in the release of the mouse oocyte from prophase I arrest PLoS One 2013 8 e53633 23326474 10.1371/journal.pone.0053633 PMC3542359

[B24] Tzivion G Avruch J 14-3-3 proteins: active cofactors in cellular regulation by serine/threonine phosphorylation J Biol Chem 2002 277 3061 3064 11709560 10.1074/jbc.R100059200

[B25] Gardino AK Yaffe MB 14-3-3 proteins as signaling integration points for cell cycle control and apoptosis Semin Cell Dev Biol 2011 22 688 695 21945648 10.1016/j.semcdb.2011.09.008 PMC3507455

[B26] Wilker EW van Vugt MA Artim SA Huang PH Petersen CP Reinhardt HC Feng Y Sharp PA Sonenberg N White FM Yaffe MB 14-3-3sigma controls mitotic translation to facilitate cytokinesis Nature 2007 446 329 332 17361185 10.1038/nature05584

[B27] Saurin AT Durgan J Cameron AJ Faisal A Marber MS Parker PJ The regulated assembly of a PKC epsilon complex controls the completion of cytokinesis Nat Cell Biol 2008 10 891 901 18604201 10.1038/ncb1749

[B28] Chen D Zhang Y Yi Q Huang Y Hou H Zhang Y Hao Q Cooke HJ Li L Sun Q Shi Q Regulation of asymmetrical cytokinesis by cAMP during meiosis I in mouse oocytes PLoS One 2012 7 e29735 22253767 10.1371/journal.pone.0029735 PMC3256179

[B29] Schultz RM Montgomery RR Belanoff JR Regulation of mouse oocyte meiotic maturation: implication of a decrease in oocyte cAMP and protein dephosphorylation in commitment to resume meiosis Dev Biol 1983 97 264 273 6189752 10.1016/0012-1606(83)90085-4

[B30] Liu C Liu Y Liu Y Wu D Luan Z Wang E Yu B Ser 15 of WEE1B is a potential PKA phosphorylation target in G2/M transition in one-cell stage mouse embryos Mol Med Rep 2013 7 1929 1937 23616086 10.3892/mmr.2013.1437

[B31] Davezac N Baldin V Gabrielli B Forrest A Theis-Febvre N Yashida M Ducommun B Regulation of CDC25B phosphatases subcellular localization Oncogene 2000 19 2179 2185 10822367 10.1038/sj.onc.1203545

[B32] Lindqvist A Källström H Karlsson Rosenthal C Characterisation of Cdc25B localisation and nuclear export during the cell cycle and in response to stress J Cell Sci 2004 117 4979 4990 15456846 10.1242/jcs.01395

[B33] Karlsson C Katich S Hagting A Hoffmann I Pines J Cdc25B and Cdc25C differ markedly in their properties as initiators of mitosis J Cell Biol 1999 146 3 573 584 10444066 10.1083/jcb.146.3.573 PMC2150562

[B34] Uchida S Kuma A Ohtsubo M Shimura M Hirata M Nakagama H Matsunaga T Ishizaka Y Yamashita K Binding of 14-3-3beta but not 14-3-3sigma controls the cytoplasmic localization of CDC25B: binding site preferences of 14-3-3 subtypes and the subcellular localization of CDC25B J Cell Sci 2004 117 3011 3020 15173315 10.1242/jcs.01086

[B35] Graves PR Lovly CM Uy GL Piwnica-Worms H Localization of human Cdc25C is regulated both by nuclear export and 14-3-3 protein binding Oncogene 2001 20 1839 1851 11313932 10.1038/sj.onc.1204259

[B36] Kumagai A Dunphy WG Binding of 14-3-3 proteins and nuclear export control the intracellular localization of the mitotic inducer Cdc25 Genes Dev 1999 13 1067 1072 10323858 10.1101/gad.13.9.1067 PMC316939

[B37] Ogg S Gabrielli B Piwnica-Worms H Purification of a serine kinase that associates with and phosphorylates human Cdc25C on serine 216 J Biol Chem 1994 269 30461 30469 7982962

[B38] Margolis SS Walsh S Weiser DC Yoshida M Shenolikar S Kornbluth S PP1 control of M phase entry exerted through 14-3-3-regulated Cdc25 dephosphorylation EMBO J 2003 22 5734 5745 14592972 10.1093/emboj/cdg545 PMC275402

[B39] Margolis SS Kornbluth S When the checkpoints have gone: insights into Cdc25 functional activation Cell Cycle 2004 3 425 428 15020839

[B40] De Souza CP Ellem KA Gabrielli BG Centrosomal and cytoplasmic Cdc2/cyclin B1 activation precedes nuclear mitotic events Exp Cell Res 2000 257 11 21 10854050 10.1006/excr.2000.4872

[B41] Jackman M Lindon C Nigg EA Pines J Active cyclin B1–Cdk1 first appears on centrosomes in prophase Nat Cell Biol 2003 5 143 148 12524548 10.1038/ncb918

[B42] Krämer A Mailand N Lukas C Syljuåsen RG Wilkinson CJ Nigg EA Bartek J Lukas J Centrosome-associated Chk1 prevents premature activation of cyclin-B-Cdk1 kinase Nat Cell Biol 2004 6 884 891 15311285 10.1038/ncb1165

[B43] Meek SE Lane WS Piwnica-Worms H Comprehensive proteomic analysis of interphase and mitotic 14-3-3-binding proteins J Biol Chem 2004 279 32046 32054 15161933 10.1074/jbc.M403044200

[B44] Pozuelo Rubio M Geraghty KM Wong BH Wood NT Campbell DG Morrice N Mackintosh C 14-3-3-affinity purification of over 200 human phosphoproteins reveals new links to regulation of cellular metabolism, proliferation and trafficking Biochem J 2004 379 395 408 14744259 10.1042/BJ20031797 PMC1224091

[B45] Hogan B Constantini LE Manipulating the mouse embryo: a laboratory manual 1986 Cold Harbor Laboratory Press, New York

[B46] Zhang Z Su WH Feng C Yu DH Cui C Xu XY Yu BZ Polo-like kinase 1 may regulate G2/M transition of mouse fertilized eggs by means of inhibiting the phosphorylation of Tyr 15 of Cdc2 Mol Reprod Dev 2007 74 1247 1254 17342725 10.1002/mrd.20703

[B47] Gallicano GI McGaughey RW Capco DG Activation of protein kinase C after fertilization is required for remodeling the mouse egg into the zygote Mol Reprod Dev 1997 46 587 601 9094105 10.1002/(SICI)1098-2795(199704)46:4<587::AID-MRD16>3.0.CO;2-T

